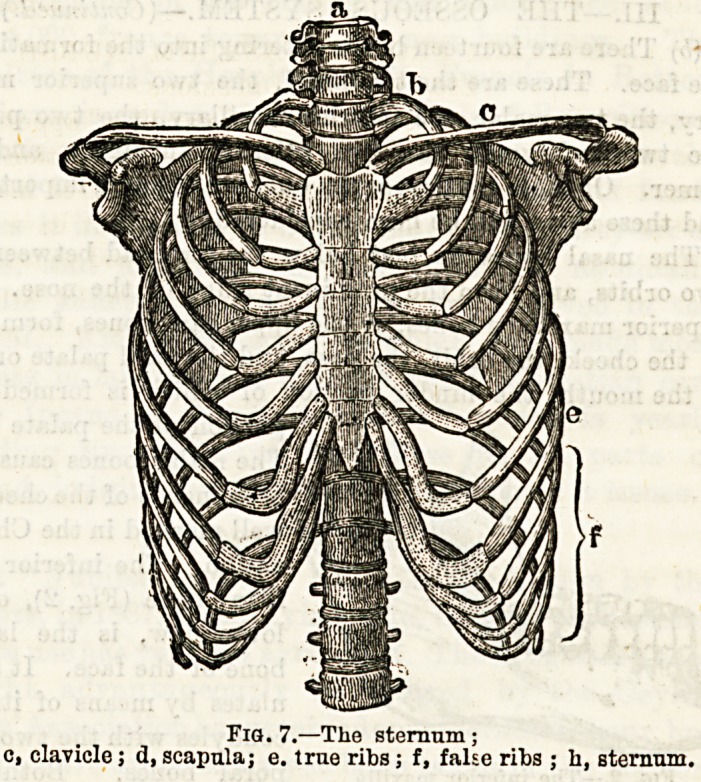# The Hospital Nursing Supplement

**Published:** 1895-01-26

**Authors:** 


### "The Hospital" Nursing Mirror.

Being the Extra Nursing Supplement of "The Hospital" Newspaper.

[Contributions for this Supplement should be addressed to the Editor, The Hospital, 428, Strand, London, W.C., and should have the word "Nursing" plainly written in left-hand top corner of the envelope.]

### News from the Nursing World.

### THE LONDON HOSPITAL.

An annual treat is provided for the Sisters at the London Hospital by Mr. John Hampton Hale, chairman of the House Committee. He evidently believes in the wisdom of making other people happy in the way most acceptable to themselves, for he places dress circle tickets and a cab at the service of all the ward sisters in turn. They go, in parties of four, to theatres selected by themselves. Thus, whilst the pantomime attracts one quartette, the Lyceum or Savoy is patronised by the next. Mr. Hale is to be congratulated on the liberality and kind thought which provides reasonable recreation for hospital workers, apart from their everyday surroundings.

### MATRONS UNDER THE POOR LAW.

For years the Workhouse Infirmary Nursing Association has endeavoured to convince the Local Government Board and the Guardians that the sick poor can only be efficiently tended by trained nurses directly supervised by a trained matron. The standing of the latter should be as a matter of course quite different to that of the matrons of old days who were not infrequently chosen from inmates of the workhouse. The position of matrons under the Poor Law was the subject chosen for the conference held under the auspices of the Matrons' Council on 17th inst. Miss Mollett reading a paper, and Mrs. Bedford Fenwick (chairman), Miss Catherine Wood, Miss de Pledge, Miss Wyld, Brigadier-Surgeon Pringle, Dr. Harris, Dr. Toogood, and others taking part in the subsequent discussion. Most of the ladies considered that authority over the nursing and domestic staffs should rest entirely with the matrons, whilst the Medical Superintendents tended to an adverse opinion, evidently considering that matrons should only be trusted to act independently so long as they happened to please the men who were legally their superior officers, however inexperienced in all practical domestic matters. It had been said of a matron's position in a Poor Law infirmary that "the only thing she could do with impunity was—nothing!" Those speakers who expressed themselves at this conference as satisfied with the existing Poor Law, owned somewhat illogically that it was easily evaded by judicious management of the Medical Superintendent. In this way it is obvious that authority is often secured by the tactful woman as a favour, instead of being the right now deemed desirable. All present appeared to believe that the Local Government Board would not be appealed to in vain, and that better times were in store for matrons. The W.I.N.A., whose aims Miss Wilson eloquently explained, deserves all honour for indefatigable efforts, sustained for many years, to gain reasonable concessions for their nurses.

### GENERAL HOSPITAL, BIRMINGHAM.

At the Birmingham General Hospital, very successful Christmas entertainments were provided for the patients and staff, the entire cost attendant on the festivities and ward decorations being covered by a fund raised for the purpose by friends of the institution. A fine tree gave much pleasure to the juvenile patients, some eighty in number, and the wards were all seasonably adorned with considerable taste, the hospital chapel being particularly pretty.

### A NOVEL PARTY.

Amongst the many Christmas entertainments given this season, an interesting one is reported from Harpurhey, where a branch of the Manchester and Salford Sick Poor Nursing Institution is established. The matron, assisted by the nurses, gave a party to all the patients they had attended in the course of the year, over two hundred responding to the kind invitation. An excellent tea was enjoyed, followed by a nigger performance, given by St. Oswald's Juvenile Troupe, and other friends.

### QUEEN'S NURSES.

The Stamford Branch of Queen Victoria's Jubilee Institute is doing excellent work, and since its establishment many other branches have been formed in the neighbourhood. General cases of a serious character have been efficiently nursed, as well as several of diphtheria, all of which did well. The Queen's Inspector gave a gratifying report after her official visit to Stamford last year.

### GRATEFUL PATIENTS.

A trained district nurse was instituted at Chagford four years ago, and her work amongst the poor appears to be highly appreciated. A committee of working men recently started a subscription, which resulted in 150 persons joining in a testimonial, and on Christmas Eve Nurse Anderson received a purse of money and an album inscribed, "Presented to Mrs. Anderson, together with a purse of money, by the working classes of the parish of Chagford, in recognition of the valuable services rendered to their families by her as nurse."

### DARLINGTON.

The position of the Darlington branch of the Queen's Jubilee Institute is excellent, the annual report showing a good balance in hand and a famous record of work. Mrs. Arthur Pease was re-elected president of the local association at the recent meeting, at which it was stated that 10,000 visits had been paid during the year by the Queen's nurses at Darlington.

### NURSE OR WASHERWOMAN?

The nurse employed by the Aylesbury Guardians has need, according to the local Press, to possess the qualifications of a laundress as well as those of a nurse. The Board asserts ignorance of the fact that the matron and nurse helped with the washing. There was some discussion at a recent meeting of Guardians as to whether actual aid or supervision of the laundry was demanded of the nurse. Apparently no inquiry was made as to the provision for the helpless or sick inmates whilst their attendant was claimed by the exigencies of the washtub! We venture to think that the sooner the Board place the sick poor under the charge of a qualified nurse and put soiled linen into the hands of competent washerwomen the better for all concerned. The invidious declaration that a nurse is to make herself "generally useful" under the matron (who sometimes, according to the Press report, has "to get up the wash"), relegates the nursing of the sick to a very secondary position.

### A COTTAGE HOSPITAL.

From Barry Dock to Cardiff has long been acknowledged as an undesirably trying journey for victims of the numerous accidents occurring in the former district. In the absence of a nearer hospital, no other course has been hitherto possible, but the difficulty will shortly be met by the establishment of a cottage hospital. Mr. Meggitt has generously placed certain premises at the disposal of the Nursing Association for a term of five years, to serve as a temporary hospital, trusting that a permanent one may be established in the town before the end of that period. The value of the nurses has been so frequently mentioned in connection with Barry Dock, that there seems little doubt of such a practical auxiliary as a cottage hospital meeting with appreciation.

### PRIVATE NURSING.

A lecture on private nursing was given at the offices of the Royal British Nurses' Association on the 18th inst. by Mrs. Gray. It was followed by a discussion, in which the details of uniform, fees, work, rest, and age were talked over. With regard to the last, more than one speaker called attention to the obvious absurdity of nurses being required to give up three or four years to training, which is not probably completed until twenty-eight or later, when at thirty-five these experienced certificated women are told they are too old for the staff of nursing institutions. On the other hand, nurses already well passed the condemned age related pleasant experiences of their continued success and of the appreciation they enjoy from their well-established private connections, thus showing that the public does not consider women in the prime of life less suitable for private nursing than their younger and less experienced sisters. The fashionable fancy of dressing nursery maids in nurses' uniform was strongly condemned, and the employers' explanation that "it gives such a nice appearance" hardly seemed to condone the injustice.

### CREDITON SICK NURSE.

The Crediton Sick Nurse Association presented an excellent report and financial statement last week at the annual meeting. The nurse, Miss Hassall, appears to be most popular with her patients, and she and the hon. secretary, Mrs. Budge, were cordially thanked for valuable services rendered during the past year. Lady Audrey Buller was elected president of the Association, and the Countess of Portsmouth, Lady Shelley, and Mrs. H. Ferguson Davie, vice-presidents for the ensuing year.

### DUTCH PROGRESS.

The Society for Rendering First Aid in Accidents proposes to give free lectures in the University at Amsterdam. Lectures on nursing are now given at Deventer, where special attention is given to district nursing. The Deaconesses Institute at Utrecht has received from the Queen and Queen Regent of Holland a subscription of 500 francs, and among the other gifts 4,000 francs towards the new buildings. The committee of the Hospital of St. Francis in Rotterdam have issued a circular to their fellow-citizens, entreating help, and pointing out that whereas the hospital is intended for sixty patients, lack of funds renders it impossible to admit more than thirty-six at a time, and although the institution is nominally Catholic adherents of all creeds are received in the hospital. Among the societies which have made most progress in late years is that for the "Christian care of the Insane in the Netherlands," which is yearly extending its sphere of usefulness in all parts of Holland, judging by the favourable reports it issues.

### NURSES IN CEYLON.

The "Jubilee money" collected in Ceylon by the Planters, in 1887, is still lying idle, because no decision as to its use has yet been arrived at. The suggestion that it might advantageously be utilised by the Ceylon Nurses' Association has received considerable favour, but no steps have apparently yet been taken in the matter. Nurses Coxall and Shankland seem to be kept busy at Hatton, and Nurse Vicary and Sister Lucie are doing good work at Colombo, whilst Nurse Linwood is attached to Polwatte, and makes her headquarters at St. Margaret's Home. The Ceylon Nurses' Association could find employment for more nurses. One is already gone out, and others would doubtless soon follow if the "Jubilee money" or other funds put the Association in a position to incur the expenses attendant on getting thoroughly trained private nurses from England.

### SHORT ITEMS.

Four demonstrations on Sick Room Cookery are arranged for the medical students at Edinburgh Royal Infirmary. They are given by teachers from the School of Cookery and Domestic Economy, and take place in the large theatre, the fee paid by each student being extremely moderate.—The report presented at the fifth annual meeting of the Scottish Association for the Medical Education of Women showed a considerable improvement in its financial position.—The St. Austell Nursing Association is working satisfactorily, the services of the two nurses being highly appreciated.—Brechin Victoria Nursing Association has also a good record, Miss Lyon, the Queen's nurse, being greatly valued in the district.—The Gateshead Nursing Association possesses a convenient home for the three district nurses who are doing good work in the neighbourhood.—A poor old man's sudden death at Stepney Workhouse was followed by an inquest at which it was shown that there is no resident medical officer, and also that a complaint of indisposition had failed to receive proper attention.—The *Strand Magazine* contains a pleasantly written article on the Royal Free Hospital.—The Tunbridge Wells Nursing Association, which supplies three trained nurses to the sick poor, is thoroughly appreciated in the district, and the Home in Limehill Road, opened last year, has proved a decided boon.—The Scottish Needlework Guild has forwarded £4 and 138 garments to the Blairgowrie Nursing Association. — The Laundry Superintendent of the Bath Workhouse has been made Assistant Matron.—The third annual meeting of the Radstock Sick Nursing Society took place last week.

### Elementary Anatomy and Surgery for Nurses.

By W. McAdam Eccles, M.B., B.S., F.R.C.S., Lecturer to Nurses, West London Hospital.

III.—THE OSSEOUS SYSTEM.—(*Continued.*)

(*b*) There are fourteen bones entering into the formation of the face. These are the two nasal, the two superior maxillary, the two malar, the inferior maxillary, the two palate, the two lachrymal, the two inferior turbinate, and the vomer. Of these the first seven only are of much importance, and these alone will be here considered.

The nasal bones lie just below the forehead between the two orbits, and form the prominent ridge of the nose. The superior maxillary bones, or the upper jaw bones, form part of the cheek, the orbit, the nose, and the hard palate or roof of the mouth, the hinder portion of which is formed by a portion of the palate bone. The malar bones cause the prominence of the cheek, so well marked in the Chinese nation. The inferior maxillary bone (Fig. 2), or the lower jaw, is the largest bone of the face. It articulates by means of its two condyles with the two temporal bones. Both the upper and the lower jaw present sockets for the teeth, which in the adult are thirty-two in number, but in the child there are but twenty temporary teeth. Each upper jaw carries two incisor, one canine, two bicuspid, and three molar teeth, and in that order from the centre outwards. Each half of the lower jaw has just the same number and in the same order, the only difference being that the molars of the upper jaw have three fangs, two placed on the outer edge of the arch, and one on the inner, while those of the lower jaw have only two roots.

Fig. 2.—The inferior maxilla.

The hyoid bone is placed in the upper part of the neck, and is shaped somewhat like an expanded U. Before passing to the bones of the vertebral column, the skull as a whole deserves some attention. When all its component bones are articulated the cavity so formed, containing the brain, is said to have a vault or roof, and a base or floor. The two deep, pyramidal sockets seen in front are the orbits or spaces in which the eyeballs are received. The roof of each, formed by part of the frontal bone, is very thin, as may be judged by its being translucent when held up to the light. The openings of each nostril, both in front and behind, should be studied with regard to their size and shape. The bones in the adult are firmly and immovably united together by sutures, which in most cases are serrated. The chief of these are: (1) The coronal, between the frontal and both parietal bones, and running transversely; (2) the sagittal, between the two parietal bones, passing directly backwards; (3) the lambdoid, like the Greek letter λ, between the occipital and both parietals; (4) the squamous, between the squamous portion of the temporal bone, and the lower edge of the parietal bone. (See Figs. 3 and 4.) In the infant the bones are separated by spaces devoid of bone, and where several of the component bones meet then this space is much increased, and is spoken of as a fontanelle. The most important one of these can be readily made out in any child under a year old, and through it can be felt the pulsation of the brain. This is evidently a weak spot in the cranial roof until it becomes closed in by bone between the first and second year of life.

Fig. 3.—Top view of the skull.

Fig. 4.—Side view of skull.

### The Vertebral Column and Thorax.

Leaving the bones of the head, we now have to take a rapid survey of the bones forming the *vertebral column*, or spine. Here will be found a beautiful adaptation of nature to the requirements of function. The spine, while acting as an adequate support, has to be freely moveable, and is therefore made up of numerous segments which are termed vertebræ. These are arranged in four groups, viz.: seven cervical or neck vertebræ, twelve dorsal or thoracic vertebræ, five lumbar or abdominal vertebræ, the sacrum and coccyx. The sacrum consists of five portions, which in the adult have become fused into one bone; so likewise the coccyx was originally in four pieces, which constitute a primitive tail. Each vertebra consists of a body in front and an arch behind, the latter enclosing a cavity through which passes the spinal cord.

Fig. 5.—Front view of skull.

There are certain outstanding processes attached to a vertebra; at the apex of the arch is the spinous process (the greater number of which can be easily felt), at the sides the transverse processes, which in the dorsal region help to support the ribs; besides these there are two pairs of articular processes—one superior, the other inferior. Each body is superimposed upon another, but between the two is a pad of elastic tissue called an intervertebral cartilage, which serves as a buffer when shocks are transmitted through the spine. The first cervical vertebra is called the atlas, because it supports the globe of the head. Between this and the occipital bone, as has already been stated, the "nodding" movements of the head take place. The atlas presents no body, its place being taken by a projection from the next vertebra, called the axis, which forms a toothlike prominence, the odontoid process. It is around this that the head, together with the atlas, rotates when moved from side to side. The process is held in position against the anterior part of the atlas by means of a band passing transversely behind it. If it should slip out of the ring so formed the spinal cord will be subjected to sudden pressure, and instant death will result, as is evidenced in cases of "broken neck." The sacrum is a very strong wedge-shaped bone, fitting in between the two hip bones. The vertebræ, when articulated together, after infancy present a series of four curves. The first is a short one in the cervical region, having its convexity forwards; the second in the dorsal region, with the concavity forwards; the third in the lumbar region, with once more the convexity forwards; and lastly, a short marked curve of the sacrum and coccyx, with the concavity forwards. (See Fig. 6.)

FIG. 6.—Vertebral column—lateral view.
1-7, bodies of cervical vertebræ—8-19, bodies of dorsal vertebræ — 20-24, bodies of lumbar vertebræ—AA, spinous processes—B B, articular surfaces of transverse processes for the tuberosities of the ribs—C, articular surface of sacrum.

The thorax has a bony and cartilagious wall, for, besides the dorsal vertebræ behind, the ribs with their costal cartilages, are placed at the sides, and the sternum, or breastbone, in front. The sternum is a long, flat bone, easily divisible into three parts; the upper, to which is attached the clavicle, or collarbone, and the cartilage of the first rib, together with part of that of the second rib, the middle portion joined to the first portion at a distinct angle, has the rest of the cartilage of the second rib, and the cartilages of all the other true ribs, attached to it, the lowest or third portion usually remains cartilaginous till late in life. (See Fig. 7.) The sternum greatly assists in protecting the organs within the thorax. The series of ribs includes twelve pairs. (See Fig. 7.) Seven of these constitute what are called the true ribs, that is to say, they not only articulate posteriorly with the dorsal vertebræ, but in front are attached through the intervention of their costal cartilages to the sides of the sternum. The remaining five are styled false ribs, and of these three, viz., the eighth, ninth, and tenth have their cartilages attached to the cartilage of the ribs above them, while the eleventh and twelfth are only attached to the vertebræ behind, and are termed free or floating. All the ribs are placed in a sloping plane from behind forwards, so that when raised in the act of inspiration the cavity of the chest is greatly increased. The thorax has an upper opening bounded by the first dorsal vertebra, the two first ribs with their costal cartilages, and the upper piece of the sternum. Through this pass various structures from and to the neck. The thorax is shut off below

FIG. 7.—The sternum;
c, clavicle; d, scapula; e. true ribs; f, false ribs; h, sternum.

from the abdomen, or belly, by a muscular partition known as the diaphragm, in which, however, there are important openings.

### Nursing in Japan.

THE central branch of the Order of the Red Cross at Berlin has granted a sum of M.10,000 to the Japanese branch of the same Order towards the relief of Japanese soldiers, wounded in China and the Corea.

Much good work has been accomplished by members of the Order in Japan. The branch was formed in the year 1877 by Vicomte Sano, ably assisted by Baron Siebold. The Vicomte is president of the society, which at first consisted only of 20 members. At the present day it has 28,000, an annual income exceeding £6,000, with a reserve fund of £40,000. The Order of the Red Cross is under the immediate patronage of the Mikado and his wife, who take a lively interest in its work, and contribute generously to the fund. The urgent need of surgical and nursing aid was forcibly felt in the revolt in the province of Sadsuma in the year 1877. After the earthquake which occurred in October, 1891, in the provinces of Owari and Mino, 70,000 persons lost their lives and 11,600 were injured, doctors and nurses were at once despatched to the scene of the disaster and ministered to more than 2,000 patients. Whereas the Japanese army is well supplied with skilful and experienced surgeons and nurses, and everything possible is arranged for the convenience and comfort of the wounded, the Chinese are singularly behindhand in this respect. No provision whatever is made for the wounded; there is not an ambulance on the field; wherever the men fall there they remain unless helped by compassionate comrades to a place of safety. In Corea the Roman Catholic missionaries have done their utmost to meet the spiritual and bodily needs of the wounded. Indeed, in several cases they have procured the erection of hospitals in different towns in Corea. Foremost among these is the hospital at Söel which Bishop Corfe, formerly Naval Chaplain to H.R.H. the Duke of Saxe-Coburg, was chiefly instrumental in establishing, as well as the English Mission in the same town.

In 1891, a large hospital was opened at Tokio by the Red Cross Society. It is built upon the model of the University Hospital at Heidelburg. Immediately upon war being declared with China voluntary assistance was offered on all sides, and numbers of male attendants followed the Japanese army to Corea. There they are doing much good work, not only among their countrymen, but also in alleviating the sufferings of their adversaries, the Chinese.

### Asylum News and Nursing.

[Contributions to this section should be addressed to THE EDITOR, 428, Strand, W.C., and have the words "Asylum News" written in left-hand bottom corner of the envelope.]

### LETTERS FROM AN ASYLUM NURSE.

### ESCAPES AND DELUSIONS.

I NOTICE that a "nurse of five years' standing" appears to be very doubtful as to the condition of the organisation of the asylum from which the patient to whom I referred in my last letter escaped. She also thought that it must have been a small asylum. As a matter of fact it happened to take place in one of the largest asylums in England. I do not think that the fact that no nurse is allowed to leave the premises without a written order can be taken as evidence of a high standard of organisation. It suggests locked doors and high airing court walls, and seems to place the staff somewhat in the position of the patients in the old bad days. The asylums in which I have worked were certainly in the first rank, and yet I never knew any such red tapeism. It would be interesting to hear the experiences of other nurses.

After this digression I will resume my story. From the accounts of the escapes given, I received some hints which were useful to me, and saved me, on more than one occasion, from being well chaffed by my fellow nurses. A charge nurse told us that she missed a patient one morning, and after searching vainly for her, high and low, reported her as having escaped. There was no sign whatever to give any clue as to how she had left the asylum. Messages were sent to the places which it was thought she might visit, and members of the staff went out to search the grounds and neighbourhood, but without success. The next morning she appeared, to everybody's great astonishment, among the other patients; declared she was very hungry, and asked if she had given them a good fright? She then, with great good humour at the trick she had played, showed where she had been. She was accustomed for some reason or other to sleep on a bed on the floor in a room off the ward corridor. She had opened a part of the bed-tick after the bed was made, crept inside, and laid herself down as flat as possible, smoothing out the bed-tick, and making it appear as if undisturbed. There she lay quite happy, until, as she said, she felt too hungry to keep the joke up any longer. The bed had been made up before the woman had played this trick, and as the sheets and blankets were not in the least disarranged, no one ever suspected that she might be hidden there. Since then I have found it useful when a patient was missing in the evening to carefully search the beds of all mischievous or very stupid patients.

In a day or two I began to get interested in my work, and the fright of my first experiences wearing off, I determined to stick to it. It took me some time to get accustomed to looking after and bathing the dirty cases. There is nothing like having such work to do, to teach one "that prevention is better than cure," and that the trouble spent in seeing that these cases are regularly attended to and taken to the lavatory is, in reality, time and labour saved. It was soon evident to me that there was as great a diversity of character among the insane patients as among people outside, and I was surprised to find how much there was to admire and respect about many of them.

I had one or two experiences with regard to delusions which were instructive. One woman had the idea that she had a mouse inside her. I first attempted to argue with her that this was absurd and impossible. The only result of this was that she got irritated with me, and had no scruples in telling me how little she thought of my intelligence. Then what I thought a brilliant idea struck me! I got a dead mouse one morning, placed it in her chamber, showed it to her, and assured her that she need not worry any more because she had got rid of her trouble. She however, informed the doctor, on his visit, that she was in a worse state than ever, because the mouse in her stomach had been breeding, and she called me as a witness that she had actually passed one from her bowels! She was most triumphant, and rated the doctor severely for not having believed her story before. All I gained was a severe wigging for having by my fancied clever (?) conduct increased and confirmed the woman's delusion. I was told that in future, while I was perfectly justified in letting the patient see that I believed her ideas were really delusions, yet I must not venture on any such experiments, nor was I to argue with patients on such subjects.

[However large our correspondent's experience of asylums may be, she is evidently unaware that the passes to which she objects have for years past proved most satisfactory in many well-managed general hospitals.—ED. *T. H.*]

### FALSE ACCUSATIONS AND UNFOUNDED COMPLAINTS BY THE INSANE.

Those engaged in nursing the insane are especially liable to suffer from the above. Fortunately for the workers in asylums, they have peculiar facilities for disproving the charges, though even they at times find it difficult. A lie that is half a truth is the blackest lie of all, and the truth may consist in correct details of surrounding circumstances, thus giving an appearance of reality to the complaint. Any lunatic may develop this unpleasant propensity, but there are some forms of insanity in which it may be looked for and guarded against. There are grumblers and complaining individuals among the insane as well as among the sane. But apart from these, the patients most likely to make groundless charges, or with only a very trifling cause for the complaint, are those suffering from hysteria, epileptics, those with delusions of persecution, and some senile cases. Sometimes it is a marked feature in the insanity of pregnancy, and in that condition in which the patient, though apparently able to talk sensibly, resists everything done for him. This phase is occasionally very prominent in puerperal cases.

Patients with hallucinations of hearing may fancy that the nurses say insulting things, and make complaints about them. The epileptic's complaint generally refers to personal violence, or the misappropriation of her property. Hysterical women are apt to charge men with indecent language or behaviour. They should be especially watched when any workmen are about. Women suffering from the insanity of pregnancy may cause great mischief by their unfounded charges against their husbands or others who have been about them. It is not the very insane who are the most dangerous. It is rather the individuals who magnify trifling things into insults who are to be guarded against. A thoughtless word, or a hand laid upon a patient's arm becomes a torrent of abuse, or a violent assault, when complained of. The only means by which the nurse can protect herself against such accusations, are vigilant observation, careful handling, and full and accurate note taking of every occurrence. A nurse should not attempt to exercise force upon any case singlehanded, but should always call another to her assistance.

### THE ROYAL EDINBURGH ASYLUM, MORNINGSIDE.

Notable and noteworthy among asylum magazines is the "Morningside Mirror." It is by no means of recent origin, having already reached the forty-ninth volume. The last number gives a very graphic description of the opening of the new Craig House by the Duke and Duchess of Buccleuch.

It is an interesting fact that no workman has lost life or limb by accident during the five years' building operations. The good work done by nurses in hospitals and elsewhere is mentioned in very complimentary terms, and hopes are expressed that more of this class may take to mental nursing. Hitherto the experiment of trying hospital nurses in asylums has not been encouraging, probably on account of the unsuitability of the nurse employed, as well as the vast change in the conditions under which she works.

This little magazine is vigorous and healthy. It is a good indication of the way in which the zeal and intellectual activity of the medical superintendent pervades the whole of the Royal Edinburgh Asylum.

DERBY BOROUGH ASYLUM, ROWDITCH.

An examination for the Medico-Psychological Nursing Certificate was held at this asylum by Dr. Macleod, of Beverley. The following candidates were successful, Wiliam Fowler, Henry Gutteridge, John Harrison, James Hendry, Mary Glenn Bostock, Mary Wright. We are glad that this good work is progressing, and also to learn that several of the nurses have joined the Nurses' National Pension Fund. The advantages of this fund should be more widely known by the staffs of asylums. It is to Dr. Macphail, the Superintendent, and his late colleague, Dr. Bruce, that we owe the thyroid treatment for some forms of insanity. For his thesis on this subject Dr. Bruce lately received a gold medal from the university of Edinburgh.

### Dress and Uniforms.

By a Matron and Superintendent of Nurses.

### Novelties in Tweed.

The Abbotsford Tweed Manufacturing Company are showing at the present time some excellent materials, suitable for costumes and cloaks. Every one admits that a really good Scotch tweed is cheapest in the end, since it is made to wear and not merely to sell, as is too often the case with inferior articles. Pure wool only, forms the composition of all these goods, so they will be found to be as warm as they are durable. These tweeds are all made in a wide width, and the price per yard is extremely moderate. There are also some very nice serges of extra thickness for winter wear, which are to be highly recommended for cloaks and skirts. For the convenience of customers who prefer to buy things ready-made, there is a special department where an easy system of self-measurement has been instituted, and an excellent fit guaranteed. This firm offers special facilities to farmers and others who breed their own sheep, in the preparation and manufacture of the raw material into any description of woollen article that may be required, merely charging a price according to the nature and finish of the cloth.

### A Sanitary Sock.

Messrs. Foster and Co. (Newry, Ireland) are to be congratulated on their "Sanita" socks, which are of excellent shape and finish, being manufactured out of pure, undyed wool. These goods will be found invaluable to those who suffer from tender feet or who are in the habit of taking active exercise, as there is nothing to chafe or irritate the most sensitive skin. They are likewise soft and pliable, thus rendering them additionally useful to those who have gouty or rheumatic tendencies. We strongly recommend a trial, which we believe will be justified by the results, and as the price is very reasonable, no one will be inclined to reject them on that score. The usual reduction is made on taking a quantity.

### The New Corset.

Since the meeting of the Anti-Corset League in the beginning of December, the subject has engrossed no inconsiderable share of public attention. Whether the League will be successful in inculcating the principles it so earnestly advocates remains yet to be seen. The platinum anti-corset which they recommend is an ingenious speciality of Messrs. Herts, Son, and Co. (Limited), Cripplegate Buildings, and is constructed on the soundest hygienic principles. It is supported from the shoulders, and secures a graceful outline to the figure without undue compression on any part. It is extremely comfortable, and has the further advantage of being fitted with bones which are warranted not to break and can easily be removed when the corset requires washing. Before condemning the principles of the League, which, after all, are based on sound common sense, it might be well to give the "Anti-Corset" a trial in order to decide whether it is not the most desirable way of securing the necessary support.

### Harrod's Stores.

The recent extension of premises at this popular emporium in the Brompton Road has resulted in the further development of its various departments. These are so complete in their nature that customers now possess the advantage of accomplishing all their purchases, from grocery to linendrapery, in the same establishment. The convenience thus ensured to that section of the public who, like the majority of our readers, have but limited time at their disposal, cannot be over-estimated. There is a promptness, too, in the attention received that is another matter for congratulation, and is certain to be appreciated where a large amount of business has to be expeditiously transacted. In the boot and shoe department, a very useful assortment of all shapes and kinds adapted for ward and general use is kept in stock. Those described expressively as "the soft and silent" are of excellent quality in every way, and most comfortable to the wearer; the heel is square and low and clamped with indiarubber, while a strap over the instep supports the foot. There are also hygienic boots and shoes to be had, and at the most reasonable prices. Soft-lined velvet and felt slippers commend themselves for night wear, and a delightful novelty in snow boots, lined throughout with flannel, and daintily finished off round the ankles with soft brown fur, claims more than a passing glance this cold weather. For the convenience of nurses a large supply of aprons in various sizes is kept. Especially noticeable are some in white union neatly hem-stitched all round. The caps are particularly well made and in good style. The "Sister Grace" and the "Sister Mabel" are shapes which are always becoming and for which there will always be a demand. They are kept in position by a string run in at the back which unties when required, and the cap without further trouble becomes flat for washing. There is an excellent assortment of dress materials, and all at the most reasonable prices per yard. A washing fabric in pale blue linen, with narrow white lines at intervals, is particularly inviting. There is also a goodly assortment of navy blue linens and drills with others too numerous to mention. The serges and other woollen materials quite come up to the general average, and can be obtained in all colours. The box department also merits a visit. There are displayed all sorts and qualities of trunks. The question of luggage is always an important one to a private nurse, and to such we would recommend the advisability of taking as light a box about as possible. Our own experience is entirely in favour of a black canvas cabin trunk, as it can be conveniently stowed away on a brougham or under the seat of a dog-cart, an important consideration to those in the country, who have to drive sometimes considerable distances to and from a station. Neither must the servants, who have to carry luggage up and down stairs, be altogether overlooked in the matter.

### Metropolitan Asylums Board—South Eastern Hospital, New Cross.

### THE NURSES' HOME.

On Thursday, 17th inst., this Home was formally opened by Captain C. W. Andrew, Chairman of the Committee of Management, and the nurses have this week moved into the luxurious quarters prepared for them. In addition to the committee, many visitors and the principal officers of the hospital were present at the opening, and as many nurses as could be spared from the wards. The history of the hospital was pleasantly given by Captain Andrew in the course of a short address, and afterwards the officers and nurses presented their chairman with an excellent portrait of himself, a copy of which is hung in the new sitting-room. The hospital can accommodate about 450 fever patients, and is one of the largest under the control of the Asylums Board. The quarters devoted to the nurses have hitherto been most inadequate and unsuitable, and the committee resolved about two and a half years ago that a proper Home must be provided. Competitive designs were invited, and that of Messrs. Quilter and Wheelhouse, 1, Adam Street, Adelphi, was accepted.

The Home consists of two separate buildings, one for the day and the other for the night staff. For the latter there are 34 separate bed-rooms, a sitting-room for the night sister, good baths, and other sanitary arrangements, and the passages are warmed by hot-water radiators.

The larger building where the day nurses sleep contains 48 separate bed-rooms, and a handsome dining-room with bay windows. The furniture of the dining-room is of solid oak, and is well proportioned to the lofty apartment which, like the sitting-room, is for the use of day and night nurses. Both rooms are 25 feet by 25 feet, and charmingly planned; in fact they are suggestive of some fine old country mansion, although few of the latter can boast of as prettily-designed windows. In the sitting or drawing room the furniture is of American walnut, upholstered very comfortably. Folded screens of polished wood, with light panels, and a good piano help to complete a most attractive interior. A sight of these fine rooms would cause considerable surprise to those who think the environments of fever nurses are as plain as the external walls which encircle the property of the Metropolitan Asylums Board.

The bed-rooms are 12 feet by 9 feet, with the exception of one or two, which architectural exigences have made somewhat larger. Each contains a combination dressing chest and washstand of satin walnut, with a marble slab for the basin, a small table and lounge chair, besides the bedstead. There is a fireplace in each room, and the shelf and overmantel are designed of iron, coloured to match the walls. Box-room, housemaids' cupboards, and hanging cupboards for the nurses' outdoor raiment have all been thought of, and appear well arranged. Whilst admiring the elegance of the woodwork, we are fain to regret that the architect has overlooked the danger of ledges becoming resting-places for dust and dirt. By banishing some and rounding off other portions of ornamental woodwork little would be lost in effect, and considerable saving in labour would be gained. At present all is bright and spotless, and there is certainly much to praise and little to criticise in this latest addition to nurses' homes. All the floors are of polished Austrian oak, and the staircase, of fireproof cement, is cased in polished oak. The hall is wide and light, and the staircase is extremely handsome, with carved balustrades and rounded polished hand-rail.

The dietary of the nurses is liberal and varied, and the appurtenances of the table leave nothing to be desired. Afternoon tea is served in the sitting-room; the pretty little pots hold "tea for two," and mount guard over cake and bread and butter. The uniform provided for fever nurses includes not only dresses, aprons, and caps, but collars, cuffs, and over petticoats; and as all the washing is done in the laundry reserved for the officers, it is evident that nurses under the Metropolitan Asylums Board are treated with great liberality.

The kindly and courteous administration of the Medical Superintendent, Dr. MacCombie, is ably seconded by the Matron, Miss Ambler Jones, who shares the Doctor's satisfaction in the improved accommodation now secured to the nursing staff.

### The Government Civil Hospital, Hong Kong.

### II.—INSIDE THE HOSPITAL.

Attached to each floor is a small ante-room for the sister's use, and a large closet, fitted up with cupboards and shelves for the patients' clothing, ward requisites, &c.

The first ward we entered was devoted to the Indians, chiefly those in Government employ—Sikhs and Mahommedans, the former recognisable by their long hair screwed up into a top-knot on the crown of the head; the latter by their short cut locks, presenting an almost shaven appearance. Some wore gay scarlet turbans wound round their heads, and all were clad in the comfortable white flannel suits provided by the hospital. All the interior walls are colour washed, skirted with a deep, dark dado, the floors of polished teak, and the beds flanked by neat lockers, made of the celebrated Canton blackwood. The wards are well-lighted by numerous glass doors, which however stand open, to admit as much air as possible during the warm weather. The Indians, like the Chinese, have but two meals a day, which consist when they are on "full diet" of curry and Indian cakes. They appreciate this more than the rice and eggs of "low diet."

The Sikhs will only eat and drink what is cooked and brought to them by a member of their own sect. The Mahommedans refuse animal food not killed by a coreligionist.

The Chinese ward resembles the other, only that the beds are furnished with mats and bamboo pillows instead of mattresses, &c. Communication between the sisters and the patients is partly carried on by means of the attendants, who interpret, using "Pidgin English," such as "No belong my" (that does not belong to me); "No, can" (that will not do); "No savey" (I do not know, &c.).

A large number of the native cases are fevers or accidents, or certain results of a free use of the Chinese chopper, a favourite weapon for general application in their quarrels.

Proceeding to the second floor, which is entirely European, I found the wards much the same as those below, only lighter, airier, and with an even more charming outlook. A few good engravings adorned the walls, and a liberal supply of books, from the hospital library, showed that there was no neglect in providing food for mind as well as body.

The smaller of the two wards was principally occupied by members of the European police force, whilst the other was filled with seafaring men of all nationalities.

The sight of a harmonium called forth the information that a weekly service is held in the ward for all those who care to avail themselves of it. It is conducted alternate weeks by the chaplain of the Seamen's Church and the colonial chaplain. The milk, which was just then being given round, was delivered to the patients in glass-stoppered bottles containing a pint or half-pint, as the case might be, morning and afternoon, and as milk is not very plentiful in Hong Kong the hospital is fortunate in obtaining such a large supply from one dairy. The Chinese do not, as a rule, care to drink milk, but prefer congee (rice-water) and tea, of which they partake very freely, hot or cold, at any hour of the day.

The doctor, or his assistant, makes a round of the wards twice a day, when all orders for the patients are given. The sister undertakes the temperatures, medicines and dressings, and general supervision, whilst the attendants look after the patients, keep the wards in order, and distribute the food, and a coolie is responsible for lavatories, verandahs, &c.

D. Y. B.

### An English View of American Nursing.

By a Colonial Matron.

After a comparatively brief experience of America I cannot do full justice to the subject, but feel it may interest my English sisters to wander with me through a few of the hospitals and charitable institutions of this continent, and make comparisons between familiar home methods and those that prevail here; the chief difference in the system of nursing being that no sisters or heads of wards hold permanent posts, the nurses in charge being undergraduates, who for the last few months of their training have care of wards. To the English mind this may seem an undue responsibility for those young in the work—a vision of waste in food, in ward appliances, and in dressings, perhaps even patients suffering from want of experienced care comes before one. This, however, seems to be all avoided by close supervision by the directress of nurses, by great adaptability and perhaps by the better education of Americans. The desire of the nurse to stand well in her class has also weight, and the system that was a necessity in the early days of nursing in this country has been found to answer well. It is economical, and the increased responsibility certainly gives the graduating nurse more self-reliance, developed judgment, and experience in controlling others than can be acquired by English nurses in the same period of training.

Nurses often enter the training schools at twenty years of age, though I believe older candidates have the preference. The course at present extends over two years. Each year's work is followed by examinations; the first year's subjects are usually anatomy, physiology, and materia medica; diseases, surgery, obstetrics, hygiene, sanitation, &c., occupying the second year.

The graduating ceremony of each class is celebrated by public bestowal of the diplomas gained, addresses of congratulation and exhortation to the graduating nurses, and general festivities; after which the class leaves to make room for the incoming pupils. The graduates usually enter their names upon the registry of their school as private nurses. They occupy rooms in the vicinity of the hospital, pay an annual registry fee of from 5 dols. to 10 dols. to the school, and receive from the school, through the superintendent of nurses, the calls for private nurses which are made there; the system of a staff of trained nurses resident in the hospital or nurses' home and sent out to private cases I have not met with here at all. Some few hospitals send out their nurses in training during the last three or six months, the fees going to the hospitals; but this is not approved of generally.

The graduate nurse receives, of course, her own fee, which may be, in New York, from 20 dols., 25 dols., to 30 dols. per week, male patients, infectious cases, and dementia calling for the higher charges. Some training schools control the charges made by the nurse. One school in Chicago, a city where fees are usually high, limits its nurses to 18 dols. per week. This limitation is much objected to by the graduates, and will probably be discontinued. It, however, has its value by preventing newly-graduated nurses from receiving the same remuneration for their services as those of longer experience. The older nurses usually make their own connection in families and with physicians, and are independent of the registry. A foreign nurse has great difficulty in securing an appointment since, in most hospitals, only nurses in training are employed, and a stranger has little chance of obtaining private cases until the supply from the schools is exhausted, and this difficulty grows greater every year as the numbers of graduates increase. There are some schools on whose registry nurses from other schools may enter, but these are more frequently met with in the Western cities than in this section of the country.

Nurses are forming themselves into alumnæ associations for mutual benefit and sympathy, and aim at possessing their own club-rooms where residence together can be had, and professional interest and standing be maintained; this will be difficult to effect by the effort of nurses alone, unless aided by friends of their school. The fees received seem high, but expenses are large, and work can by no means be relied upon all the year round, summer being always a quiet season and, as a rule, a healthful one. Training schools are often placed under the control of a separate committee of management, who elect their superintendent and vest all power in her.

Other schools are under hospital management. Where the interests of hospital and training school conflict, as in the case of charity hospitals, where politics are allowed to interfere in the administration, the School Committee is often driven to contend step by step for the rights of the probationers.

Almost all American hospitals take paying patients at rates varying from 3 dols. to 20 dols. or more per week; these fees include board, drugs, and ordinary nursing attendance.

Patients paying under 8 dols. or 9 dols. per week usually receive the free attendance of the medical staff; while those for whom better accommodation is provided, pay their medical fees as they would in their own homes.

One hospital, with a large number of private patients, gives the lowest estimated cost at 1 dol. 27 cents per day, or 8 dols. 89 cents per week; this estimate shows how this class of patients, while considering they pay for their care and maintenance, are, in fact, largely indebted to the charity of the hospitals.

The American hospitals are, as a rule, of later construction than those I am familiar with in Great Britain, and have the advantage of the latest improvements in elevators, clothes shafts, and plumbing. Electric appliances are used for lighting, ventilating, and even in some cases for heating and for motor force. The employment of tiles and marble for wainscots and flooring in place of wood, and the use of aseptic furniture in operating rooms and wards are great gains, giving increased facilities for surgical cleanliness.

Canada is not behind in good hospital accommodation—the Victoria Hospital for Sick Children in Toronto, the Royal Victoria at Montreal, and the new wing of the General Hospital, Montreal, are examples of modern construction, and leave nothing to be desired.

Of the New York hospitals, scarcely one pleased me more than the Sloane Maternity, a small obstetric hospital for fifty patients, which has white marble or white tiled floors throughout, and appears to be complete in every way. It is a gift to the city from Mrs. Sloane, *née* Vanderbilt. Two other important institutions are due to the Vanderbilt family, namely, the "Vanderbilt Clinic" and the College of Physicians and Surgeons adjoining it. Immediately opposite the latter is the Roosevelt Hospital, attached to which is the Syms operating theatre, probably unique of its kind. The total cost of the construction and equipment of this building was 200,000 dols. of the bequest, and accumulated interest, leaving 150,000 dols. to be invested for the maintenance of the building.

### Where to Go.

Sanitary Institute, Margaret Street, London.—Four lectures on Domestic Hygiene at 3 p.m. on Tuesdays and Fridays, commence March 26th.

Courses of educational lectures on Anatomy, Physiology, Hygiene, Practical Nursing, Domestic Economy, and Ward Management will be given at 17, Old Cavendish Street, commencing February 4th.

### French Schools for Trained Nurses: Their Origin and Organisation.

### II.—GRADES AND DUTIES OF MALE AND FEMALE NURSES.

BEFORE describing the three municipal schools in Paris for male and female nurses, it will be well to set forth clearly the duties of the said nurses when appointed to positions in the hospitals. The hospital nursing staff is composed of two sections; first, the male and female superintendents and sub-superintendents with their *suppléants* and *suppléantes;* second, male and female nurses. The superintendents fulfil the same duties as the sisters of charity, and replace the trained sisters in the hospitals.

These different grades in the Paris Hospital nursing staff are made manifest by outward and visible signs, each grade having its special costume.

1. The *surveillants* (male superintendents) wear a black cloth costume consisting of a short *paletot* with two rectangles embroidered in gold on the lapels. The head covering is a black cap with two gold bands above the peak, and the letters A.P. embroidered in gold; these letters signify *Assistance Publique.*

2. The *sous-surveillants* (sub-superintendents) wear the same costume as the superintendents, so far as regards shape and material, but with only one gold-embroidered rectangle on the lapel, but also with the letters A.P. on the lapel. The cap has a gold chin-strap and the letters A.P. embroidered in gold.

3. *Suppléants* wear a bluish-black cloth costume of a much coarser quality; the rectangles on the collar lapels are braided in red or blue. The cap is only ornamented with the letters A.P. embroidered in gold.

4. *Première infirmièr* (head male nurse) wears the same costume as the *suppléant*, with only one rectangle on the collar.

5. The first-class male nurses and others wear a blue cloth costume of the same description as that already described without any rectangle on the collar lapel. The cap is also blue, the letters A.P. being worked in red.

6. The *surveillantes* (female superintendents) wear a black gown and black silk caps. Full dress costume is the addition of a black silk fichu (crossed kerchief) with black silk fringe.

7. The *sous-surveillante* (sub-superintendent) wears the same costume, with the distinguishing mark of a narrow white band round the black cap.

8. The *suppléantes* wear a black bodice and skirt and a white cap with a transverse band of black ribbon, finished with a bow, and ends at the back of the cap. Full dress costume is formed by the addition of a white muslin fichu trimmed with lace.

9. The *première infirmière* (or ward nurse) wears the same costume as the *suppléante*. In the morning, whilst she is occupied in scrubbing, &c., a blue and white cotton gown is substituted for the black one, with a plain white cap.

10. *Infirmière de première classe et infirmières* (first-class ward and other nurses) have two costumes, one of blue and white striped linen, to be worn whilst they are working, the other of black material; with both a plain white cap is worn. These costumes indicate the existence of a series of grades in the hospital nursing staff, ranging from the superintendent down to the ordinary nurse.

The *surveillants* and *surveillantes* (male and female superintendents) have an entire ward, or *pavillon*, under their charge. They are responsible for the inventory of the articles entrusted to the hospital by the patients being correctly made; they also make out a list of the jewellery, papers, &c., found on the patients, write the *bons* (orders for diets and extra rations), and serve the patients with their allotted portions.

The male and female superintendents begin work at six o'clock a.m., overlook what has been done, examine the report of the *suppléante de veille* (night nurse), in order to make themselves acquainted how the night was passed, and what occurred. They also ascertain the exact condition of the patients, and give them the purgatives and emetics ordered to be taken by the patient whilst fasting. They receive and serve out the milk rations, and draw up a list of the arrivals and departures of the patients during the previous evening, which is sent to the director's office. The male and female superintendents are also responsible for the correctness of the inventory of the linen given out for the use of the patients. They are likewise called upon to be thoroughly familiar with all the details of the *cahier de visite*, a register containing the directions given by the hospital physicians and surgeons in reference to treatment and diet. The duties of the sub-superintendents, male and female, differ very slightly from those of the superintendents, whom they help and replace when necessary. The sub-superintendents, both male and female, are helped by the *suppléantes*, male and female, who are called upon to overlook the night nurses, forming a kind of link between the superintendents and the nurses. The male and female nurses keep the wards clean and in order, wash and attend to the patients, change their linen, keep their beds in order, remove soiled sheets, and put on clean ones. After a certain time of hospital service the nurses are promoted by the hospital director to the grade of *premier infirmièr* or *première infirmière* when no complaint has been made against them. In due time they are promoted to the rank of first-class nurses; they are then on the way to obtain the posts of *suppléants* and *surveillants*, but unless they qualify themselves to obtain a nurse's diploma from one of the schools, already described in the preceding article, these positions are unattainable.

(*To be continued.*)

### Notes and Queries.

### Queries.

(63) *Dictionary.*—I take THE HOSPITAL every week and find it most useful and instructive, and I beg to ask advice as to whether Hoblyn's "Dictionary of Medical Terms" is useful for nurses?—*Nurse F.*

(64) *Demonstrations.*—Where can I get a full-sized model of a fœtus for demonstrations?—*Nurse B.*

(65) *Red Cross.*—Where are the Red Cross nurses trained?—*Would-be Red Cross.*

(66) *Weighing Machine.*—Where can a weighing machine be hired for use in an infectious hospital?—*Matron.*

(67) *Cottage Hospital.*—Is it usual for a *locum tenens* to be paid by the matron she replaces or by the committee of a cottage hospital?—*Matron.*

(68) *District Nurse.*—Which is the best part of Glasgow for a nurse to start in, on her own account, as district nurse and midwife to people who could afford to pay a small fee—too well-to-do to be nursed by an ordinary district nurse, but not well enough off to pay for a private nurse?—*L.O.S.*

(69) *Clinical Clerk.*—How is a position as clinical clerk secured by a woman?—*C.*

### Answers.

(63) *Dictionary* (*Nurse F.*).—It is a most useful book for a nurse to possess; the last edition is considerably enlarged. Thanks for your kindly appreciation of our efforts to help nurses.

(64) *Demonstrations* (*Nurse B.*).—Messrs. Baillière, Tindal, of King William Street, London, would procure one for you. We advise your asking them for particulars of cost, &c

(65) *Red Cross* (*Would-be Red Cross*).—The Royal Red Cross is an order conferred by the Queen, and is a distinction much coveted by nurses. It is given for distinguished service in war. Do you mean a foreign nursing association?

(66) *Weighing Machine* (*Matron*).—The hiring of anything for an infectious institution would certainly be open to objections. You must, of course, buy a machine outright. Is it patients or provisions that you want to weigh?

(67) *Cottage Hospital* (*Matron*).—We presume you mean a substitute for your annual holiday. The committee will certainly pay reasonable charges. A trained hospital nurse who wanted to have such experience would probably be content to accept a small fee, with travelling and laundry. A matron could not be expected to pay unless she guaranteed to do so when engaged.

(68) *District Nurse* (*L.O.S.*)—You should communicate with an established district nurses' association, and get reliable information as to the demand for another nurse. Are you trained for district nursing or have you only the diploma of the L.O.S. which qualifies for midwifery, not for general sick nursing?

(69) *Clinical Clerk* (*C.*).—Only by entering a medical school. You had better see the Secretary, School of Medicine for Women, Handel Street, London, W.C.

### Wants and Workers.

[THE attention of correspondents is directed to the fact that "Helps in Sickness and to Health" (Scientific Press, 428, Strand) will enable them promptly to find the most suitable accommodation for difficult cases.]

CAN anyone give me information as to how to obtain a post as sister or charge nurse in a colonial hospital?—*Nurse A.*

Will anyone give me information about the cost of living, housekeeping, &c., in Chili? Also the best route?—*Nurse.*

### Everybody's Opinion.

[Correspondence on all subjects is invited, but we cannot in any way be responsible for the opinions expressed by our correspondents. No communications can be entertained if the name and address of the correspondent is not given, or unless one side of the paper only be written on.]

### NURSING AT LAMBETH INFIRMARY.

"A Trained Nurse" writes: Will you excuse my writing a few lines regarding the paragraph in The Hospital about the Lambeth Infirmary. My sister is also a trained nurse, and I should be very sorry to think that any slur should be cast on Miss Griffiths' work, as she is a woman for whom we have a great respect, and she has done much to improve nursing at the Lambeth Infirmary during 22 years of matronship. We fail to see why any other superintendent should come between her and her nurses.

[Another letter on this subject, too long for insertion, has reached us, which speaks in equally appreciative terms of Miss Griffiths' work at Lambeth.—Ed., *T.H.*]

### PRIVATE NURSING IN SCOTLAND.

"An Enquirer" writes: Will any of the readers of The Hospital kindly give me such information about nursing in Scotland as will throw light on a case which has come under my own notice? An eminent Scottish surgeon has employed a young lady who has merely had six months' training in one of the London hospitals to nurse a patient upon whom he performed a serious operation. Surely if such an influential man is content with a nurse trained for so short a time it is quite useless for other women to spend three years in an elaborate course of preparation for private nursing.

### SPURIOUS CO-OPERATIONS AND NURSING HOMES.

"A Nurse" writes: I read with pleasure the paragraph in the last issue of your interesting and widely-circulated paper calling the attention of nurses to so-called co-operations and homes. Unfortunately these institutions exist not only in a northern city, and I earnestly entreat all who may think of joining any such to make close inquiries before doing so, and to ascertain if possible that the place has not been misrepresented in every way. By doing this nurses will avoid discomfort and loss, the latter not merely financially, but of reputation. Inexperienced nurses just leaving provincial hospitals without any private resources had better remember the old adage, "All is not gold that glitters."

### UNAUTHORISED ADVERTISEMENTS.—A CAUTION TO APPLICANTS.

"The Matron of a Large Institution in the North" writes: A short time since I advertised for a probationer in The Hospital, and from a large number of applicants selected one, and was glad to think the trouble of correspondence, &c., was over. To my surprise fresh applicants applied, stating that they had seen my advertisement in the "Nursing Record." Does it not seem most unfair to the matron, whose time is valuable, and still more unfair to the applicants (who waste time and stamps), that these unauthorised advertisements should appear, and, as in this case, *appear after the post is filled*. My answer to the "Record" applicants was to the effect that the advertisement in the "Nursing Record" was quite unauthorised, and that there was not a vacancy. You may make use of the substance of my letter, but I do not wish my name or address to appear. It may interest you to know that in answer to your advertisement I had over sixty applications. The "Record" gave us three.

### THE ABUSE OF THE UNIFORM.

"A Trained Nurse" writes: I enclose you a cutting from *Cassell's Saturday Journal*, which shows a fresh use (or shall I say abuse?) of a nurse's dress. After remarking on the courtesy generally extended to nurses, the writer refers to "the experience of two women journalists who recently visited the East End on a difficult mission dressed as nurses." Perhaps had the poor men and women suspected that they were being duped into showing respectful consideration to sham nurses, these "journalists" might have been taught a useful if unpleasant lesson. I heard an experienced nurse speak recently of another degradation to which the trained nurse's uniform is subjected. She said, "there are five women in the town where I live, four of whom are servants, all dressed in bonnets, veils, cloaks, cuffs, and other professional appurtenances, because their mistresses say 'It gives such a nice appearance.' The other woman is also untrained, but she officiates as a nurse, and she is most particular about her uniform!"

### CORRECTION.

Mr. T. Vincent Jackson writes: Permit me to say that I disapprove of the condescending reply of Miss Pauline Peter to my "correction of a correction," a reply which seems to me merely a prevarication. Miss Peter, in her "correction," made two false statements in reference to the District Nurses' Home and the district nurses of this town (Wolverhampton); these I asked Miss Peter to withdraw, and she has not done so. I cannot carry this matter further, except to express a hope that Miss Peter will endeavour to be more accurate in any communication she may in the future make to The Hospital.

### For Reading to the Sick

### PRAYER.

**Motto.**

Who goes to bed and doth not pray,
Maketh two nights of every day.
—*George Herbert*.

**Verses.**

Prayer is the burden of a sigh,
The falling of a tear—
The upward glancing of an eye
When none but God is near.—*Montgomery*.

Then fainting soul arise and sing!
Mount! but be sober on the wing;
Mount up, for Heaven is won by prayer;
Be sober, for thou art not there!—*Keble*.

They who have steeped their souls in prayer
Can every anguish calmly bear—
They who have learned to pray aright
From pain's dark well draw up delight.
Your words are fair,
But oh! the truth lies deeper still!
I know not—when absorbed in prayer—
Pleasure or pain, or good or ill;
They who God's face can understand
Feel not the motions of His Hand.—*Houghton*.

Pray! though the gift you ask for
May never comfort your fears,
May never repay your pleading—
Yet pray, and with hopeful tears!
An answer—not that you long for,
But diviner—will come some day;
Your eyes are too dim to see it,
Yet strive and wait and pray.—*A. Proctor*.

**Reading.**

When Plato gave Diogenes a great vessel of wine, who had asked but a little, and a few caraways, the cynic thanked him with this rude expression: "Thou neither answereth to the question thou art asked, nor givest according as thou art desired; being enquired of 'How many are two and two?' thou answereth 'twenty.'"

So it is with God and us in the intercourse of our prayers; we pray for health, and He gives us, it may be, a sickness that carries us into Eternal Life; we pray for necessary support for our persons and families, and He gives us more than we need; we beg for a removal of a present sadness, He gives us that which makes us able to bear twenty sadnesses, a cheerful spirit, a peaceful conscience, and a joy in God, as an antepast of eternal rejoicings in the Kingdom of God.—*Jeremy Taylor*.

A good prayer is not like a strategem of war, to be used but once. No; the oftener the better. The clothes of the Israelites, whilst they wandered forty years in the wilderness, never waxed old. So a good prayer, tho' often used, is still fresh and fair in the ears and eyes of Heaven.

Despair not, then, thou simple soul, who hast no exchange of raiment, whose prayer cannot appear every day at Heaven's Court in new clothes. Only add new, or new degrees of old affections thereunto, and it will be acceptable to God, thus repaired as if new created.—*Thomas Fuller*.

This is that which most often hindereth Heavenly consolation, that thou art too slow in turning thyself to prayer.—*Thos. à Kempis*.

## Figures and Tables

**Fig. 2. f1:**
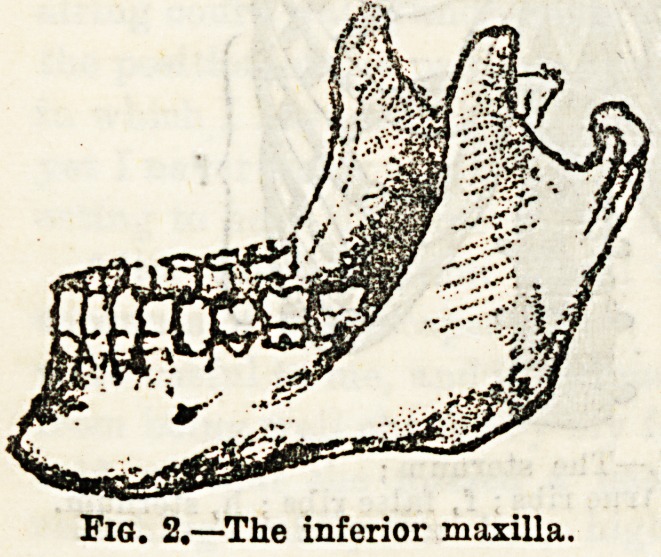


**Fig. 3. f2:**
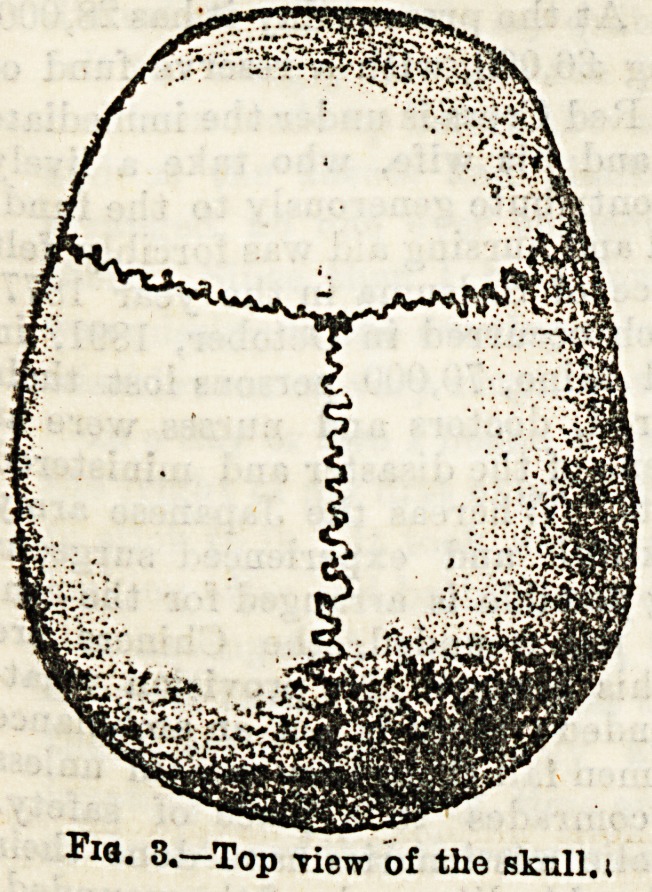


**Fig. 4. f3:**
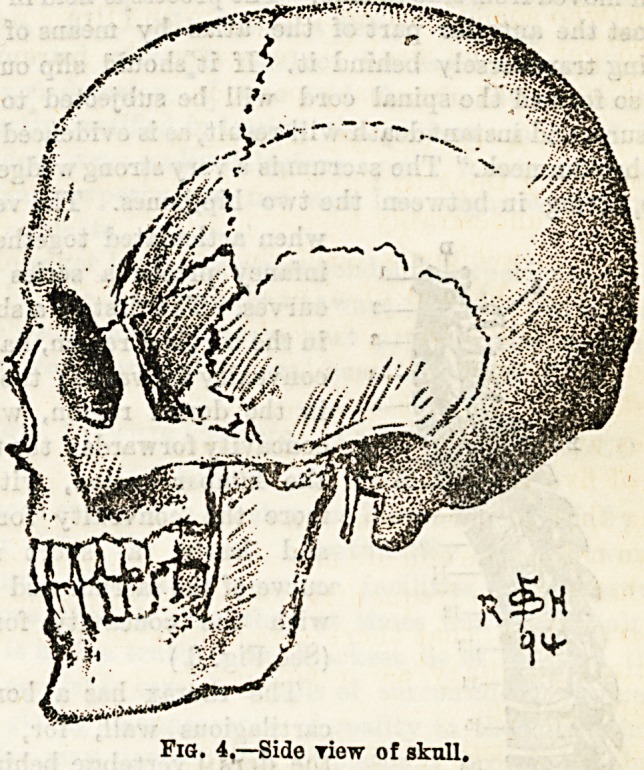


**Fig. 5. f4:**
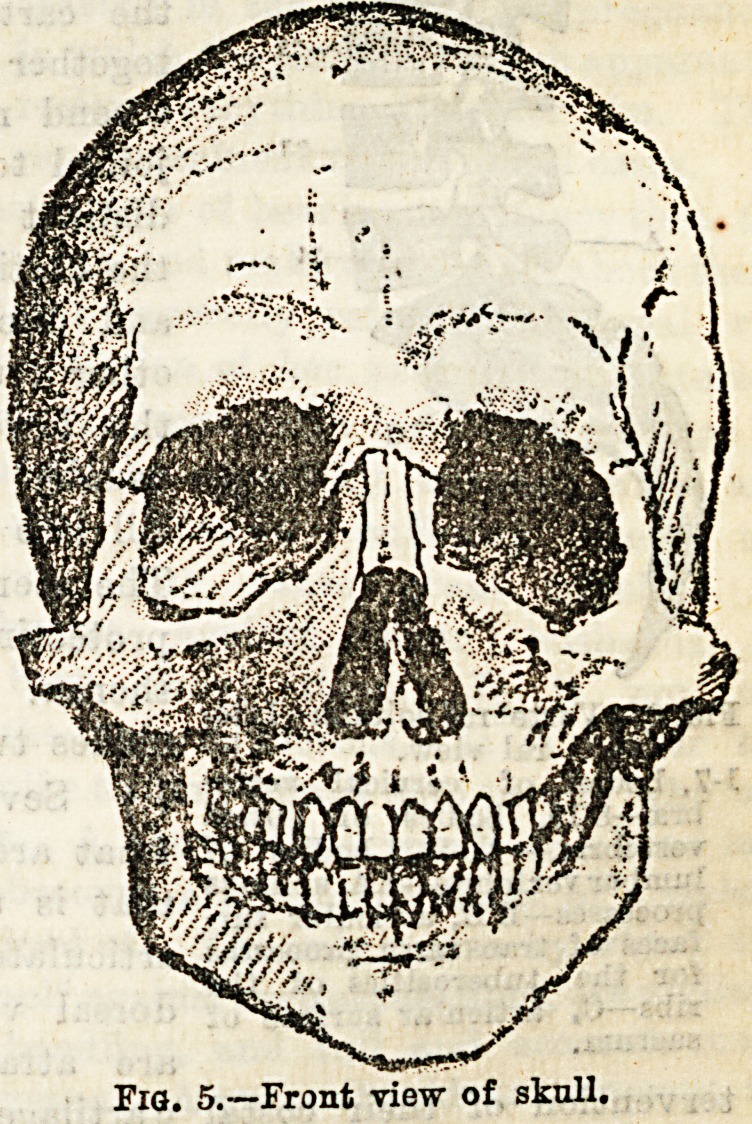


**Fig. 6. f5:**
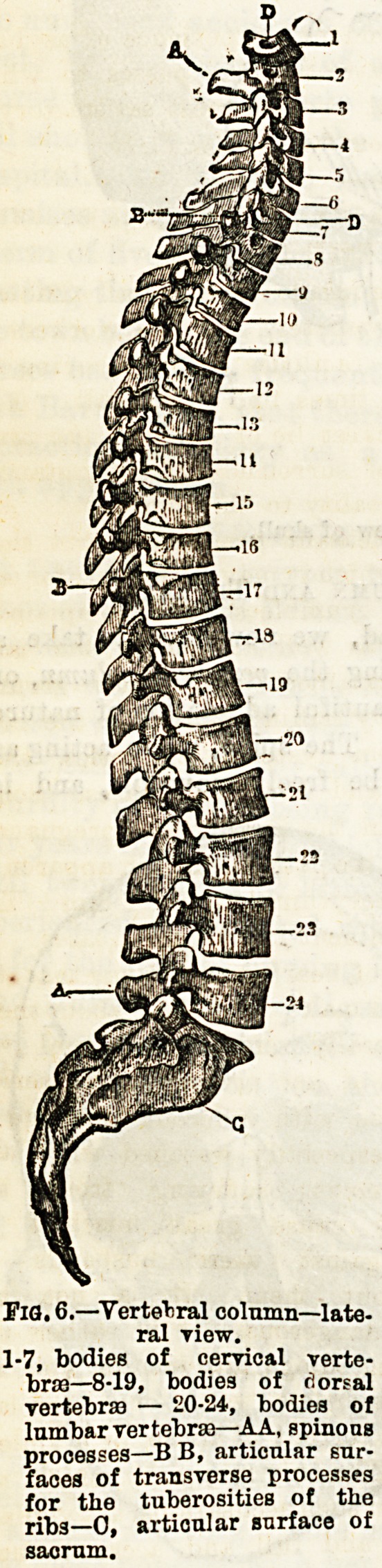


**Fig. 7. f6:**